# The predictive role of CD4^+^ cell count and CD4/CD8 ratio in immune reconstitution outcome among HIV/AIDS patients receiving antiretroviral therapy: an eight-year observation in China

**DOI:** 10.1186/s12865-019-0311-2

**Published:** 2019-08-28

**Authors:** Chong-Xi Li, Yu-Ye Li, Li-Ping He, Jing Kou, Jin-Song Bai, Jun Liu, Bo Tian, Li-Juan Cao, Kun-Hua Wang, Yi-Qun Kuang

**Affiliations:** 1 Department of Dermatology and Venerology, The First Affiliated Hospital of Kunming Medical University, Kunming, 650032 China; 2 Institute of Infection and Immunity, Henan University & Center for Translational Medicine, Huaihe Clinical College, Huaihe Hospital of Henan University, Kaifeng, 475000 China; 3 Department of HIV/AIDS, The Third People’s Hospital of Kunming, Kunming, 650041 China; 4 School of Public Health, Kunming Medical University, Kunming, 650500 China; 5 School of International Education, Henan University, Kaifeng, 475001 China; 6 NHC Key Laboratory of Drug Addiction Medicine, First Affiliated Hospital of Kunming Medical University, Kunming, 650032 China

**Keywords:** HIV/AIDS, Clinical predictor, Immune reconstitution, Prognosis, HAART

## Abstract

**Background:**

The immune reconstitution after initiation of highly active antiretroviral therapy (HAART) among HIV-infected individuals substantially affects patients’ prognosis. However, the dynamic characteristics and predictors of reconstitution outcome remain unclear.

**Methods:**

In this study, the HIV/AIDS patients with sustained virological suppression (viral load < 50 copies/ml) after HAART were enrolled. The patients were subgrouped into immunological non-responders (INRs) (< 200 cells/μl), immunological inadequate responders (IIRs) (200 ~ 500 cells/μl) and immunological responders (IRs) (> 500 cells/μl) according to the CD4 cell count after two-year HAART. The immune reconstitution data based on the CD4^+^ and CD8^+^ cell counts with 8-year follow-up were collected for analysis.

**Results:**

The CD4^+^ cell counts in the immunological responders (IRs) were significantly higher than in the immunological non-responders (INRs) and immunological inadequate responders (IIRs) (*P* <  0.001). The overall CD4^+^ cell count and CD4/CD8 ratio in the IRs increased faster than the IIRs and INRs. The CD4^+^ cell count growth at 0.5 year and 1 year after HAART in the IRs was significantly higher than the IIRs and INRs. The ROC curve demonstrated that 1 year CD4^+^ cell count had the highest predictive value, with the best cut-off value of 188 cells/μl, the predictive sensitivity was 81.0%, the predictive specificity was 85.2%, false positive rate was 14.8%, false negative rate was 19.0%, positive predictive value (IR) was 63.0%, negative predictive value (INR) was 93.5%.

**Conclusions:**

Taken together, our findings suggest that early initiation of HAART can reduce the immune reconstitution failure. The combination of baseline CD4^+^ cell count and baseline CD4/CD8 ratio may serve as a valid predictor of immune reconstitution prognosis after HAART.

**Electronic supplementary material:**

The online version of this article (10.1186/s12865-019-0311-2) contains supplementary material, which is available to authorized users.

## Background

The combination antiretroviral therapy (cART), also called highly active antiretroviral therapy (HAART), has substantially changed the lives of HIV/AIDS patients [1]. Although the HAART contains the viral replication in patients with prolonged undetectable plasma viral RNA levels, i.e. viral load < 50 copies/ml (cpm), it does not invariably show immune reconstitution [2]. Even in patients with naïve T-cell recovery, the restoration of cell number is a gradual process in the setting of complete viral suppression. Immune reconstitution following HAART is characterized by distinct CD4^+^ and CD8^+^ T cell dynamics, often displaying dichotomist trends according to disease stage [3, 4]. The dynamics of immune reconstitution under long-term HAART varies among different people and regions, and the mechanisms involved remain unclear. The HIV-1-specific CD4^+^ T cell immunity plays a dominant protective role in primary HIV infection [5]. The thymus output affects the immune reconstitution, the enhanced thymus output could benefit HIV/AIDS patients at late stage [6]. Counterturn of immunosuppression by HAART leads to exaggerated immune reconstitution inflammatory syndrome (IRIS) which seem to have started prior to treatment. Inflammatory markers, chemokines and cytokines are biological markers of innate and adaptive immune activation, which can be proved to be of clinical value after proper verification [7]. The naïve CD4^+^ cell count is the optimal reference index in prognosis of immune reconstitution [6]. However, the detection of naïve CD4^+^ cell count is not a routine work in most hospitals, especially in China. Therefore, some acceptable and useful predictive markers are needed.

In this study, a total of 280 HIV/AIDS patients in the HIV/AIDS department of the Third People’s Hospital of Kunming from January 2005 to April 2015 with sustained virological suppression were enrolled. The demographic information, clinical data, CD4^+^ cell counts and CD8^+^ cell counts under 8-year follow-up were collected for analysis of the predictor of immune reconstitution prognosis in patients.

## Methods

### Ethics statement

This study was approved by the First Affiliated Hospital of Kunming Medical University, and written informed consents were obtained from all study participants. All experiments were performed in accordance with the approved guidelines and regulations according to the principles expressed in the Declaration of Helsinki, and the experimental protocols were approved by the institutional review boards of Kunming Medical University and Henan University.

### Patients and study design

The inclusion criteria were patients underwent HAART over two years, and the viral load was sustaining < 50 cpm after two-year HAART. Enrolled patients were outpatients received HAART over two years and with sustained viral load < 50 cpm in the Department of AIDS in the Third People’s Hospital of Kunming during January 2005 and April 2015. According to the CD4 counts of two-year ART, the patients were subgrouped into immunological non-responders (INRs) (< 200 cells/μl), immunological inadequate responders (IIRs) (201 ~ 500 cells/μl) and immunological responders (IRs) (> 500 cells/μl) according to the CD4 cell count after two-year HAART. The data of all participants excluded all non-compliant patients or patients with history of treatment interruption. The viral loads of all patients were < 50 cpm, and no viral blips were observed in all patients.

Three sexually matched and age-matched groups of patients were selected at regular interval in Excel dataset, and the demographic and clinical information of these patients with follow-up from 0.5 year to 8 years, as well as the CD4 cell count, CD8 cell count, and CD4/CD8 ratio were collected for analysis.

### Laboratory measurements

About 5 ml venous blood of each patient was collected in heparin anticoagulation vacuum blood collection tube. The CD4^+^ and CD8^+^ T lymphocyte numbers and CD4/CD8 ratio were measured with the MultiTEST IMK Kit (BD) by the single platform on FACSCalibur™ flow cytometry (BD). Viral load was determined by the Virus Load Detector (Siemens).

### Statistical analysis

The datasets were established by Microsoft Excel program, and then subjected to SPSS17.0 software for statistical analysis. The numerical data with normal distribution data were expressed as mean ± standard deviation (SD). The total number was analyzed by using the random analysis of the variance. The numerical data were calculated by the χ^2^ test of the crosstab. The measurement data with skewed distribution was expressed as median (M) and range interquartile (P_25_, P_75_). The comparison before and after treatment was performed by the paired rank-sum test, the three groups were compared with a completely random design of the rank-sum test. The *P* <  0.05 was defined as a statistical significance. Multiple groups with *P* < 0.05 were subjected to further pairwise comparison; the test level was 0.05/test time. The receiver operating characteristic (ROC) curve was employed to analyze the sensitivity and specificity of predictors associated with the immune reconstitution outcome.

## Results

### Demographic characteristics of patients

A total of 280 patients out of 2564 outpatients at Third People’s Hospital of Kunming were enrolled for analysis. The average age of the 280 patients was 42.40 ± 10.47 years, and the male-to-female ratio was 2.5:1. The infection rate of hepatitis C in the INRs group (35.2%) was significantly higher than that in the IRs (15.1%) (*P* = 0.012). There was no significant difference in age, sex, marital status, infection route and hepatitis B infection among the three groups (Table 1). In addition, among 280 patients, one patient of the INRs group was diagnosed with parotid epithelial cancer at 8 years of antiviral therapy, and then underwent surgical resection without chemotherapy or radiotherapy. All the other patients had no cancers, autoimmune diseases and history of hormone usage during the observation period.

**Table 1:** The demographical characteristics of three groups

	INRs group (*n*, %)	IIRs group (*n*, %)	IRs group (*n*, %)	Total	F/χ^2^	*P value*
Age (M ± SD)	43.03 ± 10.42	42.10 ± 10.40	42.14 ± 10.74	42.40 ± 10.47	0.232	0.793
Gender						
male	69 (78.4)	82 (68.9)	49 (67.1)	200 (71.4)	3.135	0.209
female	19 (21.6)	37 (31.1)	24 (32.9)	80 (28.6)		
Time from HIV-confirmed to HAART initiation (days), M (P_25_,P_75_)	20 (11, 41)	40 (17, 298)	24 (12, 397)	27 (13, 142)	13.081	0.001
Nadir CD4 count (cells/μl), M (P_25_,P_75_)	58 (28, 91)	165 (63, 234)	290 (213, 367)	126 (52, 252)	106.482	< 0.001
Marital status						
unmarried	17 (19.3)	19 (16.0)	13 (17.8)	49 (17.5)	3.062	0.801
married	56 (63.7)	82 (68.9)	47 (64.4)	185 (66.1)		
divorced	12 (13.6)	15 (12.6)	8 (11.0)	35 (12.5)		
widowed	3 (3.4)	3 (2.5)	5 (6.8)	11 (3.9)		
Transmission route						
drug use	28 (31.8)	26 (21.8)	10 (13.7)	64 (22.9)	8.937	0.063
homosexual	4 (4.5)	6 (5.0)	7 (9.6)	17 (6.1)		
heterosexual	56 (63.7)	87 (73.1)	56 (76.7)	199 (71.0)		
HBV					0.596	0.742
Yes	7 (8.0)	10 (8.4)	4 (5.5)	21 (7.5)		
No	81 (92.0)	109 (91.6)	69 (94.5)	259 (92.5)		
HCV					8.887	0.012
Yes	31 (35.2)	28 (23.5)	11 (15.1)	70 (25.0)		
No	57 (64.8)	91 (76.5)	62 (84.9)	210 (75.0)		
Cancers	1	0	0	N/A	N/A	N/A
history of chemotherapy/radiotherapy	0	0	0	N/A	N/A	N/A
History of Immunosuppressive	0	0	0	N/A	N/A	N/A
history of autoimmune disease	0	0	0	N/A	N/A	N/A

### Dynamics of immune reconstitution

The CD4^+^ cell counts and CD4/CD8 ratios at year of 0, 0.5, 1, 2, 3, 4, 5, 6, 7 and 8 after HAART initiation among three groups were presented in Table 2. In the INRs, the CD4^+^ cell count and the CD4/CD8 ratio significantly increased during the first three years after HAART (*P* < 0.001), there were no significant differences were observed in CD4 cell count and CD4/CD8 ratio thereafter (Table 2). In contrast, in the IIRs and the IRs, the CD4^+^ cell count significantly increased during the first four years (*P* < 0.05) after HAART, and the CD4/CD8 ratio significantly increased in three years (*P* < 0.005). Interestingly, a significant increase in CD4/CD8 ratio at year 6 (compared to year 5) was observed (*P* = 0.05) in the IIRs after HAART.

**Table 2:** CD4^+^ T-cell count and CD4/CD8 ratio dynamics among three groups

Time post-HAART (year)	Case (n)	CD4^+^ count (cells/μl)^a^	χ^2^	*P* value	CD4/CD8 ratio	χ^2^	*P* value
INRs group							
0	88	52 (23,93)			0.09 (0.05,0.15)		
0.5	88	115 (82,154)	65.636	< 0.001	0.16 (0.11,0.24)	46.545	< 0.001
1	88	127 (97,176)	5.628	0.018	0.19 (0.14,0.27)	13.136	< 0.001
2	88	145 (119,173)	1.943	0.0163	0.23 (0.16,0.31)	10.227	0.001
3	87	175 (139,214)	26.793	< 0.001	0.27 (0.19,0.43)	29.897	< 0.001
4	86	191 (135,234)	2.977	0.084	0.33 (0.22,0.53)	15.070	< 0.001
5	56	185 (141,226)	0.891	0.345	0.30 (0.22,0.49)	1.786	0.181
6	37	206 (151, 264)	1.324	0.250	0.33 (0.23,0.46)	0.027	0.869
7	32	199 (142, 250)	0.032	0.857	0.36 (0.24,0.50)	6.125	0.013
8	14	210 (140,346)	0.286	0.593	0.45 (0.29,0.72)	0.286	0.593
IIRs group							
0	119	175 (74, 270)			0.17 (0.10,0.29)		
0.5	119	255 (187, 337)	49.824	< 0.001	0.31 (0.17,0.48)	85.723	< 0.001
1	119	290 (220, 393)	28.508	< 0.001	0.37 (0.21,0.56)	17.017	< 0.001
2	119	332 (271, 410)	16.407	< 0.001	0.41 (0.27,0.65)	28.508	< 0.001
3	118	366 (292, 483)	18.880	< 0.001	0.48 (0.33,0.72)	36.915	< 0.001
4	111	412 (308, 522)	3.973	0.046	0.55 (0.36,0.71)	3.252	0.071
5	75	418 (309, 501)	1.613	0.204	0.52 (0.37,0.78)	3.853	0.050
6	51	449 (348, 542)	7.078	0.008	0.57 (0.38,0.70)	1.588	0.208
7	41	456 (332, 630)	0.220	0.639	0.64 (0.47,0.77)	5.488	0.019
8	25	464 (333, 677)	1.960	0.162	0.67 (0.55,0.86)	1.960	0.162
IRs group							
0	73	314 (254, 384)			0.31 (0.25,0.38)		
0.5	73	472 (386, 587)	50.973	< 0.001	0.52 (0.40,0.83)	50.973	< 0.001
1	73	529 (460, 680)	26.889	< 0.001	0.62 (0.45,0.93)	14.918	< 0.001
2	73	631 (574, 765)	30.260	< 0.001	0.74 (0.55,1.00)	38.479	< 0.001
3	73	620 (542, 763)	2.000	0.157	0.75 (0.61,1.14)	7.247	0.007
4	70	657 (528, 818)	5.714	0.017	0.83 (0.65,1.06)	1.429	0.232
5	46	656 (569, 737)	3.130	0.077	0.81 (0.63,0.94)	1.391	0.238
6	26	646 (491, 805)	0.000	1.000	0.85 (0.72,1.00)	2.462	0.117
7	18	640 (488, 841)	2.000	0.157	0.79 (0.71,1.15)	0.222	0.637
8	10	687 (569, 801)	0.400	0.527	0.80 (0.58,0.98)	0.400	0.527

^a^presented as median (P_25,_ P_75_)

**Fig. 1:**
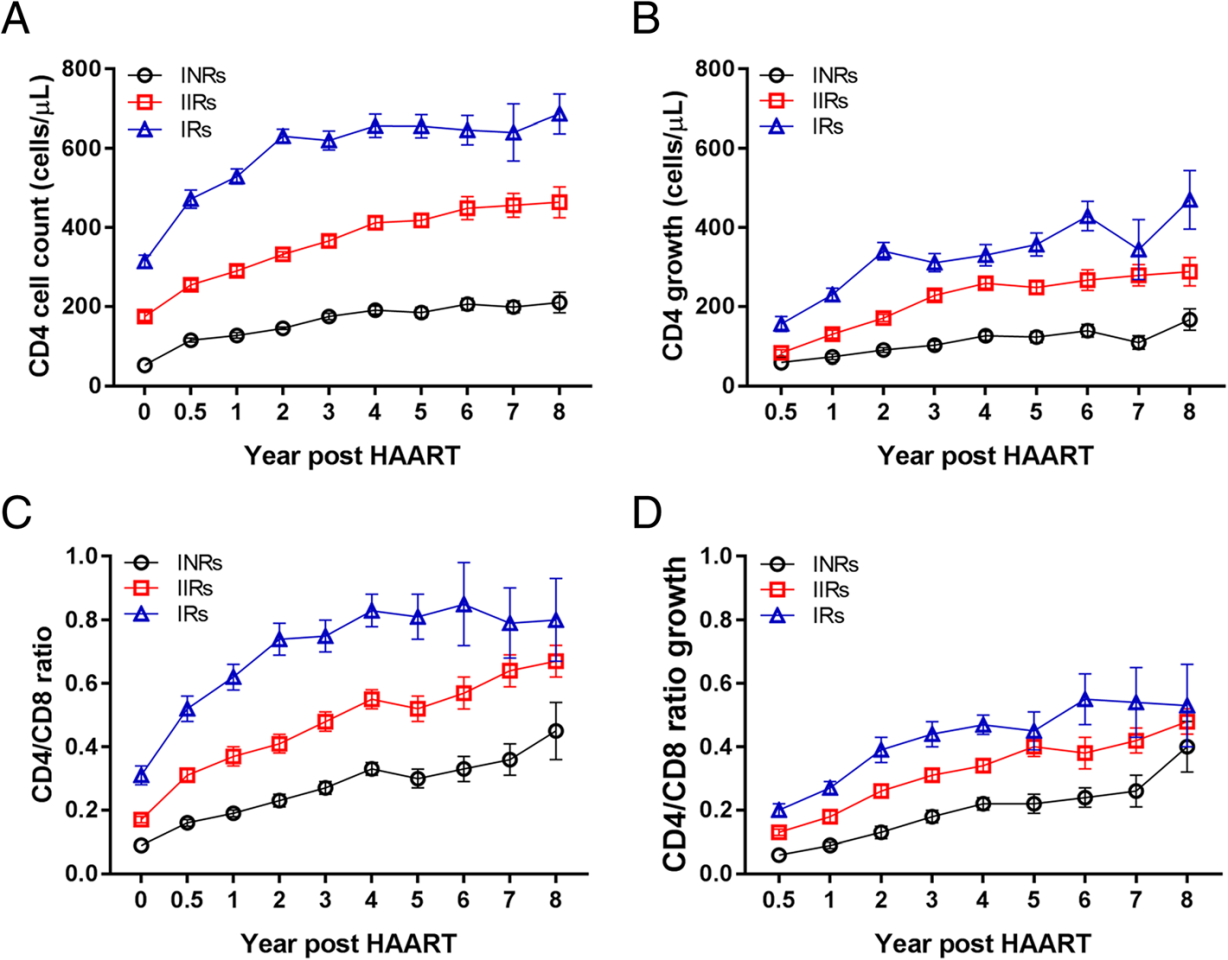
The characteristics of immune reconstitution in three groups. (**a**) The dynamics of CD4 cell counts at different time-points (from 0 ~ 8 years). (**b**) The dynamics of CD4 cell count growth at different time-points (from 0.5 ~ 8 years). (**c**) The dynamics of CD4/CD8 ratios at different time-points (from 0 ~ 8 years). (**d**) The dynamics of CD4/CD8 ratio growth at different time-points (from 0.5 ~ 8 years). The data are presented with median ± SE. The black cycles indicate the INRs, the red squares depict the IIRs, and the blue triangles indicate the IRs

The CD4^+^ cell count at year of 0, 0.5, 1, 2, 3, 4, 5, 6, 7 after HAART in IRs was significantly higher than those in the INRs (*P* < 0.001) and IIRs (*P* < 0.001) (Additional file 1: Table S1). There were significant differences between each two groups (*P* < 0.001). The CD4^+^ cell count in IRs entered a fast increase stage during two years after HAART (Fig. 1A), and then increased slowly though the cell count growth showed a steady level even a little bit decease at year 7 (Fig. 1B). However, the CD4^+^ cell count in IIRs and INRs showed slow increase all the time after HAART (Fig. 1A), and the cell count growth kept at steady increasing level except year 7 in the INRs. The CD4/CD8 ratio and CD4/CD8 ratio growth in IRs both increased during first 4 years after HAART (Fig. 1C and D), while the CD4/CD8 ratio in IIRs and INRs demonstrated relatively slow increase during the same time after HAART (Fig. 1C), and the CD4/CD8 ratio decreased at year 7 (Fig. 1C), though the CD4/CD8 ratio growth kept at steady increasing level (Fig. 1D).

### The effect of baseline CD4^+^ cell count and HAART regimen on immune reconstitution

Next, aimed to understand the effect of baseline CD4^+^ cell count and HAART regimen on immune reconstitution, a multivariate logistic regression analysis of the baseline CD4^+^ cell count, baseline CD4/CD8 ratio, HAART regimen, and HAART duration was conducted. The OR data demonstrated that, the risk of reconstitution failure in patients with baseline CD4^+^ cell count < 100, 101 ~ 199 and 200 ~ 349 cells/μl was 24.994, 12.252, 3.469 times to patients with baseline CD4^+^ cell count ≥350 cells/μl, respectively. The risk of reconstitution failure in patients with baseline CD4/CD8 ratio < 0.2 and 0.2 ~ 0.5 was 2.327 and 1.453 times to patients with baseline CD4/CD8 ratio > 0.5, respectively. The initial HAART regimen containing LPV/r was a protective factor in immune reconstitution, by which the risk of immune reconstitution failure was 0.563 times lower than those using TDF + 3TC + EFV regimen. The risk of immune reconstitution failure in patients receiving HAART < 2 years, 2 ~ 4 years and 4 ~ 7 years was 4.124, 2.344, 1.919 times to the risk in patients receiving HAART > 7 years, respectively (Table 3).

**Table 3:** Multivariate analysis of factors associated with immune reconstitution

	B	SE	Wald	OR	*P* value	95% confidence interval	
						Lower limit	Upper limit
Threshold	0.705	1.934	0.133		0.716		
	4.465	1.934	5.327		0.021		
Baseline CD4 cell count (cells/μl)							
< 100	3.219	0.224	206.140	24.994	< 0.001	16.114	38.807
101 ~ 199	2.506	0.186	181.218	12.252	< 0.001	8.506	17.649
200 ~ 349	1.244	0.161	59.899	3.469	< 0.001	2.531	4.753
≥ 350	0^a^						
Baseline CD4/CD8 ratio							
< 0.2	0.845	0.186	20.639	2.327	< 0.001	1.615	3.349
0.2 ~ 0.5	0.374	0.168	4.929	1.453	0.026	1.044	2.019
> 0.5	0^a^						
HAART regimen							
Including LPV/r	−0.573	0.286	4.012	0.563	0.045	0.176	0.988
NVP + 3TC + D4T	−0.128	0.207	0.381	0.879	0.537	0.586	1.320
EFV + 3TC + D4T	−0.261	0.306	0.723	0.770	0.395	0.422	1.404
3TC + EFV + AZT	0.098	0.183	0.284	1.100	0.594	0.770	1.579
NVP + 3TC + AZT	0.059	0.153	0.148	1.060	0.700	0.786	1.430
3TC + TDF + EFV	0^a^						
HAART time (year)							
< 2	1.417	0.248	32.776	4.124	< 0.001	2.539	6.697
2 ~ 4	0.852	0.227	14.132	2.344	< 0.001	1.503	3.654
4 ~ 7	0.652	0.192	11.570	1.919	0.001	1.137	2.795
7 ~ 11	0^a^						

^a^reference group

### ROC curve estimation of predictors

**Fig. 2:**
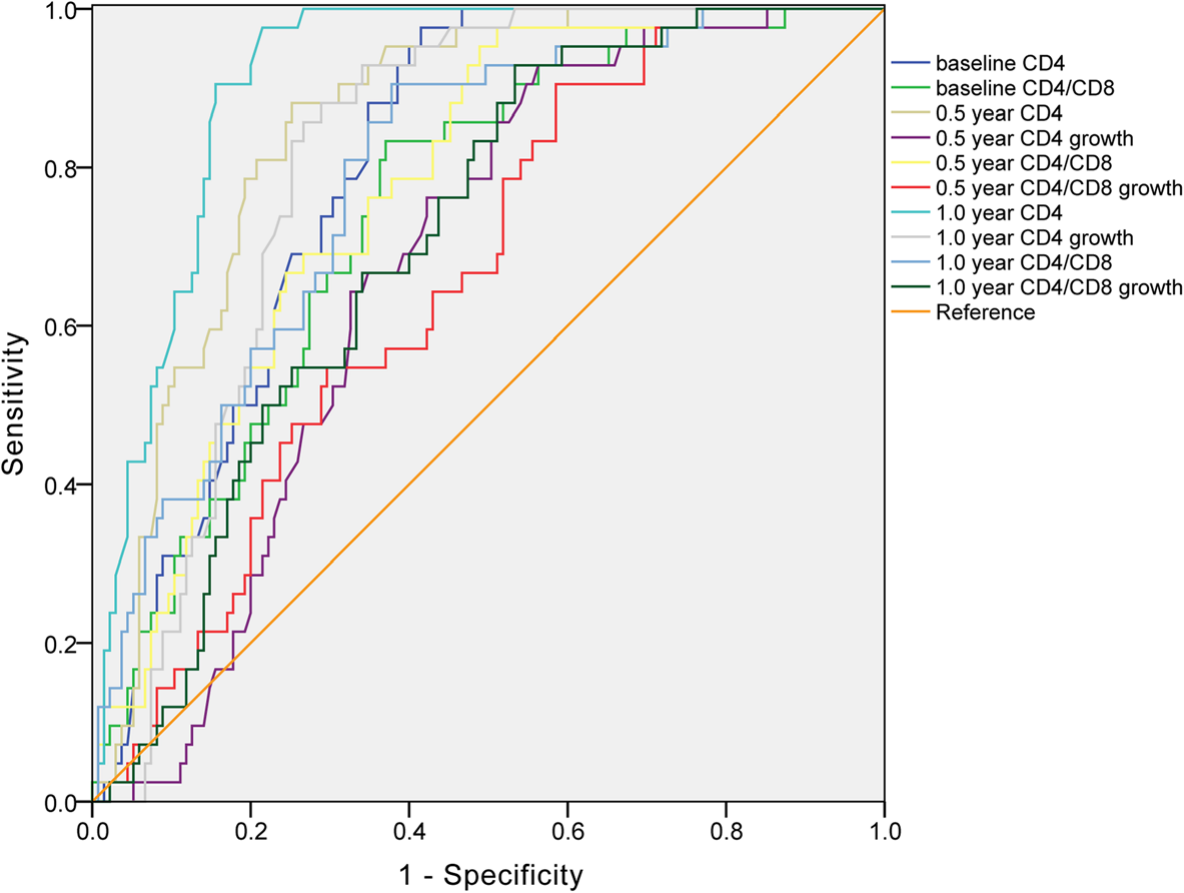
The ROC cure of predictive indexes. The X-axis depicts the specificity of predictor, the Y-axis indicates the sensitivity of the predictor. The diagonal line is the reference

The ROC curve (Fig. 2) showed that the baseline CD4^+^ cell count, baseline CD4/CD8 ratio, 0.5 year CD4^+^ cell count, 0.5 year CD4^+^ cell count growth, 0.5 year CD4/CD8 ratio, 0.5 year CD4/CD8 ratio growth, 1 year CD4^+^ cell count, 1 year CD4^+^ cell count growth, 1 year CD4/CD8 ratio and CD4/CD8 ratio growth could be the predictive indexes of immune reconstitution outcomes. The area under the curves was shown in Additional file 1: Table S2. Among them, 1 year CD4^+^ cell count had the highest predictive value, with the best cut-off value of 188 cells/μl, the predictive sensitivity was 81.0%, the predictive specificity was 85.2%, false positive rate was 14.8%, false negative rate was 19.0%, positive predictive value (IR) was 63.0%, negative predictive value (INR) was 93.5%. The best diagnostic cut-off point for baseline CD4^+^ cell count was 90 cells/μl, the predictive sensitivity was 71.4%, the predictive specificity was 71.1%. The predictive cut-off point of baseline CD4/CD8 ratio was 0.15, the predictive sensitivity was 76.2%, the predictive specificity was 63.7%.

By using the combined baseline CD4 < 90 cells/μl and 1 year CD4 cell count <188 cells/μl to predict the 5 year INRs after HAART, the results demonstrated that the sensitivity, specificity, false positive rate, false negative rate, positive predictive value and negative predictive value were 69.0, 88.9, 11.1, 30.0, 65.9 and 90.2%, respectively.

## Discussion

In this study, our data showed that the baseline CD4^+^ cell count and CD4/CD8 ratio in the IRs were significantly higher than that in the INRs and IIRs (*P* < 0.001), while the CD8^+^ T-cell count was not significantly different. This indicates the pertinent role of the baseline CD4^+^ cell count in immune reconstitution [5, 6, 8]. The high baseline CD4/CD8 ratio (> 0.5) was associated with immune reconstitution success, which is in accordance with previous reports that the critical role of CD4/CD8 ratio normalization [9, 10]. Regardless of CD4^+^ cell count during HAART treatment, frequent virological monitoring might be necessary to reduce the risk of virological failure [11].

The CD4^+^ cell count in the IRs grew fast than those in the INRs and IIRs groups after HAART (Fig. 1). The growth of CD4 count and CD4/CD8 ratio in IRs were both higher than the other two groups. The growth of CD4^+^ cell count and CD4/CD8 ratio in the IRs were significantly higher than those of the INRs and IIRs at 0.5 year, 1 year, 2 years, 3 years and 4 years after HAART. This suggests that slow CD4^+^ cell count growth after HAART treatment is prone to cause immune reconstitution failure in patients. The CD4^+^ cell count reaches plateau stage one year ahead in INRs, which is a predictive signal of immune non-response and implies timely adjuvant therapy to improve immune response.

It has been shown that low baseline CD4^+^ cell count strata entered a low level of plateau, while high baseline CD4^+^ cell count entered a high level of plateau [12]. In this study, among the three groups with baseline CD4^+^ cell count ≤100 cells/μl, the CD4^+^ cell count growths in IRs at 0.5 year and 1 year after HAART were higher than in INRs and IIRs. It implies that the CD4^+^ cell count growth at 0.5 year and 1 year could be the predictors of immune response, which is consistent with previous report [13]. Furthermore, the linear model regression analysis showed that the slopes of CD4 count growth during 0 to 2 years and 3 ~ 8 years after ART among INRs, IIRs, and IRs were significantly different (Additional file 1: Table S3). It implies that the CD4 count growths among three groups are significantly different. The CD4 count of IRs increased the most during first two years. Although the CD4 counts of INRs and IIRs groups increased relatively slow during first two years, it could be elevated under sustained ART, as we can see of the 3 ~ 8 years data (Additional file 1: Table S3 and Fig. 1), which is in accordant with previous report [14]. It has been shown that ART drugs are associated with immune dysfunction in HIV-1-infected subjects [15, 16]. However, as the lack of the cell functional data except for the cell counts, it is hard to interpret the absolute role of CD4 count in immunological reconstitution.

ROC curves showed that the 1 year CD4^+^ cell count was of diagnostic ability as predictors of the immune response after treatment. The sensitivity and specificity are superior to the combination of baseline CD4^+^ cell count and baseline CD4/CD8 ratio, which was reported unable to be a predictive immune reconstruction of poor indicators [17]. In addition, the combination of CD4 count and CD4 count growth after one-year HAART is an option in evaluation of clinical prognosis of immune response. These findings contribute to the early prediction of immune response in a timely manner to take adjuvant therapy to improve the immune function of patients.

The limitation of our research is the relative small sample size in certain group of poor immune responders. With the larger sample size, the predictive role of the baseline CD4^+^ cell count and CD4/CD8 ratio in immune reconstitution will be more clear, which calls for more research in future. The other limitation is that we do not have the numbers of the subgroups of CD4^+^ T-cells. Increasing evidence showed that different subtypes of CD4^+^ cells affect the immune reconstitution [18, 19], and it has been reported that the percentage of baseline naïve CD4^+^ T-cells was a better prognostic factor for immune reconstitution under long-term therapy [6]. In addition, the baseline viral load is associated with the dynamics of immune reconstitution in patients [20]. The viral load pre-HAART was not analyzed in our work, and this will be considered in further work.

## Conclusions

Altogether, timely diagnosis and early initiation of HAART can reduce the immune reconstitution failure. The one-year CD4^+^ cell count after HAART initiation is a preferable predictor of immune reconstitution in HIV/AIDS patients with sustained viral suppression.

## Additional file

Additional file 1: **Table S1.** Comparison of CD4^+^ T-cell count at different time point of HAART among three groups. **Table S2.** The area under the ROC curve. **Table S3.** Regression analysis of CD4 count growth with HAART time. (DOC 53 kb)

## Data Availability

The majority of datasets used and/or analyzed during the current study are available from the indicated published resources. The remaining data, including model code, are available from the corresponding author on reasonable request.

## Abbreviations

3TC: Lamivudine
AIDS: Acquired Immune Deficiency Syndrome
cART: combination antiretroviral therapy
CD4: cluster of differentiation 4
CD8: cluster of differentiation 8
EFV: Efavirenz
HAART: Highly active antiretroviral therapy
HIV: Human immunodeficiency virus
IIRs: Immunological inadequate responders
INRs: Immunological non-responders
IRIS: Immune reconstitution inflammatory syndrome
IRs: Immunological responders
TDF: Tenofovir
