# Supplementary material for: The predictive role of CD4+ cell count and CD4/CD8 ratio in immune reconstitution outcome among HIV/AIDS patients receiving antiretroviral therapy: an eight-year observation in China
Source: BMC Immunol. 2019 Aug 28;20:31. doi: 10.1186/s12865-019-0311-2 (PMC6712592; doi:10.1186/s12865-019-0311-2)
Supplement: Supplementary file 1 — Table S1. Comparison of CD4+ T-cell count at different time point of HAART among three groups. Table S2. The area under the ROC curve. Table S3. Regression analysis of CD4 count growth with HAART time. (DOC 53 kb) [file 12865_2019_311_MOESM1_ESM.doc]

**Table S1.** Comparison of CD4^+^ T-cell count at different time point of HAART among three groups

| **Time post-HAART (years)** | **χ2** | ***P* value** |
| --- | --- | --- |
| 0 | 75.909 | <0.001 |
| 0.5 | 161.874 | <0.001 |
| 1 | 180.350 | <0.001 |
| 2 | 199.936 | <0.001 |
| 3 | 146.066 | <0.001 |
| 4 | 99.362 | <0.001 |
| 5 | 71.169 | <0.001 |
| 6 | 35.079 | <0.001 |
| 7 | 22.474 | <0.001 |

**Table S2.** The area under the ROC curve

|  | **Area** | **SE** | ***P* value** | **95% confidential interval** | |
| --- | --- | --- | --- | --- | --- |
|  |  |  |  | **Lower limit** | **Upper limit** |
| Baseline CD4 count | 0.799 | 0.032 | <0.001 | 0.736 | 0.862 |
| Baseline CD4/CD8 ratio | 0.746 | 0.039 | <0.001 | 0.668 | 0.823 |
| 0.5 year CD4 count | 0.852 | 0.029 | <0.001 | 0.796 | 0.909 |
| 0.5 year CD4 count growth | 0.672 | 0.041 | <0.001 | 0.593 | 0.752 |
| 0.5 year CD4/CD8 ratio | 0.771 | 0.036 | <0.001 | 0.700 | 0.843 |
| 0.5 year CD4/CD8 ratio growth | 0.659 | 0.043 | <0.001 | 0.575 | 0.743 |
| 1.0 year CD4 count | 0.913 | 0.021 | <0.001 | 0.872 | 0.953 |
| 1.0 year CD4 count growth | 0.808 | 0.032 | <0.001 | 0.746 | 0.870 |
| 1.0 year CD4/CD8 ratio | 0.787 | 0.037 | <0.001 | 0.715 | 0.858 |
| 1.0 year CD4/CD8 ratio growth | 0.707 | 0.040 | <0.001 | 0.628 | 0.785 |

**Table S3.** Regression analysis of CD4 count growth with HAART time

|  |  | **0 ~ 2 years ART** |  |  | **3 ~ 8 years ART** |  |  |  |
| --- | --- | --- | --- | --- | --- | --- | --- | --- |
|  | **Regression formula (y = α + βx)** | **F** | **t** | ***P* value** | **Regression formula (y = α + βx)** | **F** | **t** | ***P* value** |
| INRs | y = 105.426 + 23.011x | 13.316 | 12.950 | < 0.001 | y = 150.617 + 13.175x | 29.387 | 17.763 | < 0.001 |
| IIRs | y = 113.349 + 81.450x | 118.979 | 7.027 | < 0.001 | y = 331.508 + 25.854x | 48.039 | 25.107 | < 0.001 |
| IRs | y = 151.007 + 186.370x | 174.718 | 4.958 | < 0.001 | y = 676.769 + 2.958x | 0.166 | 27.955 | > 0.05 |

*P* value of the slope
